# Bouts of exercise elicit discordant testosterone: cortisol ratios in runners and non-runners

**DOI:** 10.20945/2359-3997000000042

**Published:** 2018-05-07

**Authors:** Thiago Paes de Barros De Luccia, José Eduardo Soubhia Natali, Alexandre Moreira, José Guilherme Chaui-Berlinck, José Eduardo Pereira Wilken Bicudo

**Affiliations:** 1 Universidade de São Paulo Universidade de São Paulo Instituto de Biociências Departamento de Fisiologia São Paulo SP Brasil Departamento de Fisiologia, Instituto de Biociências, Universidade de São Paulo (USP), São Paulo, SP, Brasil; 2 Universidade de São Paulo Universidade de São Paulo Escola de Educação Física e Esporte Departamento de Esporte São Paulo SP Brasil Departamento de Esporte, Escola de Educação Física e Esporte, Universidade de São Paulo (USP), São Paulo, SP, Brasil; 3 University of Wollongong University of Wollongong School of Biological Sciences NSW Australia School of Biological Sciences, University of Wollongong, NSW, Australia

**Keywords:** Testosterone, cortisol, physical endurance

## Abstract

**Objective:**

The testosterone:cortisol ratio (T:C) is suggested to be used in order to examine whether physical exercise generates either a “catabolic environment” or an “anabolic environment”. The present study aims to evaluate the acute time-course profile of cortisol and testosterone due to an episode of physical exercise. A biphasic profile in the T:C ratio response was hypothesized.

**Materials and methods:**

Morning sessions of treadmill running at two different intensities (Heart Rate at 65% and 80% of the maximum cardiac reserve) were performed by 6 male non-runners (NR) and 12 trained male runners (subdivided into trained runners T1 and T2). Cortisol and testosterone were measured in saliva. NR and T1 ran for 30 minutes at both intensities, and T2 ran for 46 minutes (± 4.1) at 65% and 42 minutes (± 3.5) at 80%.

**Results:**

In the 80% heart rate target, both groups of runners showed the biphasic time-profile, while the non-runners group did not. However, at the 65% level, none of the groups presented the hypothesized biphasic response.

**Conclusions:**

A biphasic time-profile in the testosterone:cortisol ratio can be seen in short-bout, high intensity exercise (treadmill running) during the morning in men trained for this specific physical activity.

## INTRODUCTION

From the classic fight-or-flight reaction to subtle dominance relationships in groups, changes are observed in specific hormonal values (1,2). Part of these changes can be understood in terms of their direct metabolic consequences, and both physical and psychological factors seem to play a role as causal factors (1). Although physical exercises are beneficial to health, such activities can generate harmful effects in both men (3) and women (4), a situation that is aggravated by excessive physical exercise (5,6). However, it is not fully known how the beneficial health and fitness-related effects of exercise come to end, subsequently becoming harmful to the human body. The study of hormonal changes related to physical exercise, which have been the focus of much research in the fields of physiology and health, may contribute to this subject (1).

Although there is still no isolated marker capable of diagnosing training problems and/or overtraining (7–9), several indicators have been proposed in recent decades in search of this supposed turning point. One of these markers is the ratio between testosterone, considered an anabolic hormone, and cortisol, considered a catabolic hormone (10,11).

The testosterone:cortisol ratio has been reported as a sign of anabolic status in athletes before competing (12), acute training response (13,14), psychophysiological responses to competition venue (15), training effects (16) and training motivation (12,17). An imbalance between the anabolic and catabolic *milieu* of the metabolism could be associated with certain components of the prescribed exercise (e.g. training volume and intensity) that should be monitored with the intention of improving sports and exercise performance whilst avoiding any deleterious effects from such activity (18).

With regards to acute hormonal responses related to exercise, the testosterone: cortisol ratio (T:C) has been suggested to be used in order to examine whether physical exercise generates either a “catabolic environment” or an “anabolic environment” (19). In a long-term training process, monitoring T:C has been advocated in order to verify the hormonal responses to a given training load, which could aid in preventing non-functional overreaching or overtraining syndrome (20). In this case, is believed that a catabolic environment might be persistently present (with elevated cortisol and low testosterone concentration) (21), associated with decline in performance, psychological changes, and neuroendocrine disorders.

In general, a low testosterone concentration may be indicative of poor health in men (22), and extreme levels of circulating androgens, whether high or low, can have negative effects on women's health (23). In women, chronic hypercortisolism has been associated with exercise-induced amenorrhea (24). In fact, the analysis of bone mineral density in this group changed the idea of amenorrhea in athletes being a benign event, and linked this phenomenon to premature bone loss associated with a decline in the levels of progesterone and estradiol (25). In a prospective, population-based study of chronic heart disease related to “stress” in men, a low ratio between testosterone and cortisol showed a strong positive association with the components of insulin resistance syndrome (26).

**Figure 1:**
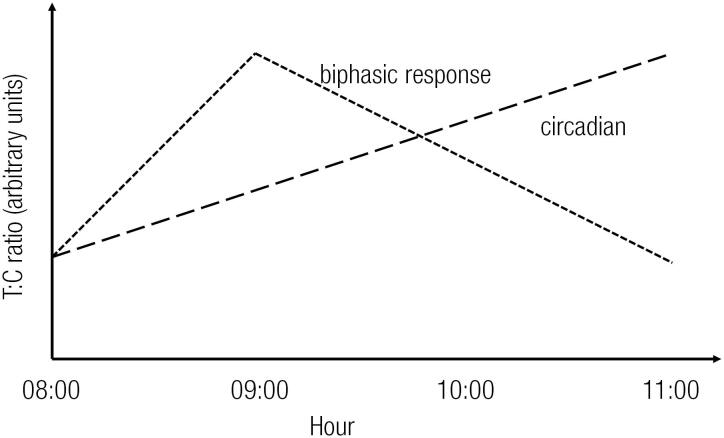
Graphical representation of the T:C ratio throughout the morning. The dashed line shows the expected physiological circadian rhythm of T:C. The dotted line represents the hypothesized biphasic response due to a bout of exercise.

According to some studies, the onset of physical activity stimulates the production of both cortisol and testosterone (27,28). However, cortisol remains elevated in the circulation following the episode of exercise, negatively affecting the synthesis of testicular testosterone (29). These events could generate a biphasic time-profile in the T:C ratio, a possible occurrence that has not yet been well studied. These events, which are considered in our hypotheses, are pictorially illustrated in Figure 1 (References used to build the chart (30–32), in which we show the expected circadian variation in T:C and the biphasic response elicited by the physical activity described above.

From this point of view, it is possible to observe that the added effect of acute exercise and the circadian rhythms of testosterone and cortisol obscures our understanding of the biphasic time-profile. Therefore, it is important to analyze the dynamics of these hormones throughout the day. A single isolated measurement might provide a poor picture depending on the sampling phase in relation to the action of cortisol. In fact, this putative biphasic time-profile could play a role in the contradictory data presented in the literature regarding the T:C ratio and acute physical activity (33).

### Questions in the study

The present study aims to evaluate the acute time-course profile of cortisol and testosterone due to an episode of physical exercise, and to answer the following questions:

1. Is there a biphasic profile in the response of the T:C ratio?
2. Is there a relationship between this profile and the intensity of acute physical exercise?
3. Is there a relationship between this profile and the duration of acute physical exercise?
4. Is there a difference in this profile between runners and non-runners?

## MATERIALS AND METHODS

### Subjects

Volunteers were selected and divided into two groups in accordance to their previous habitual physical exercise profile (the trained runner group (T, n = 12) and non-runner group (NR, n = 6)). The trained volunteers ran an average of 42 (± 19.2) km/week. They were randomly assigned to two subgroups, T1 and T2, described below. The non-runner volunteers had varied physical activities but they were not runners. Only male volunteers were selected for the study (mean age = 36 years (± 8.32); mean body mass = 68.8 kg (± 8.32). All participants had a clinical follow-up and signed an informed consent form (the study was approved by the Ethics Committee of the Bioscience Institute – University of Sao Paulo, CAAE 12937713.0.0000.5464).

### Preliminary data collection

Volunteers visited the laboratory for the first time in order to be physically evaluated and receive instructions about the experimental procedures. Subsequently, each volunteer performed a running session on the treadmill to establish the individual speed corresponding to the target heart rates (see below).

### Exercise intensity zones – target heart rates – intensity versus demand

The exercise intensity was determined by the heart rate reserve method (34):


(1) $$R_{p}=R_{rest}+p\times (R_{\max}-R_{rest})$$

Where R_rest_ is the resting heart rate, R_max_ is the inferred maximum heart rate of the subject (220 – age in years), p is the desired percentage of the maximum intensity (heart rate reserve) and R_p_ is the computed target heart rate.

The protocols used were differentiated by the exercise intensity, the training level of the subjects, and a controlling variable (time or heartbeat) that limits the duration of the exercise. The protocols for each subset are summarized in Table 1.

**Table 1:** Schematics of the experiments

	Fitness	Non-runners	Trained	Trained
	Protocol	Fixed time	Fixed time	Fixed heartbeats
Intensity	65%	NR R_65_	T1 R_65_	T2 R_65_
	80%	NR R_80_	T1 R_80_	T2 R_80_

The experimental groups were separated into subsets with regards to training level (trained runners vs. non-runners) and the protocol (fixed time vs. fixed heartbeats). Individuals from these subsets performed the exercise at two intensities (65% vs. 80%).

The two exercise intensities selected were 65% and 80% of the heart rate reserve (R_65_ and R_80_, respectively). During the experimental procedures, treadmill speed was adjusted every 5 min based on the mean heart rate observed within this 5 min period in order to keep the heart rate as close as possible to the desired target zone.

Each running session for volunteers belonging to the NR or T1 groups comprised a 30-min run at the individually predetermined R_65_ and R_80_. Therefore, these groups performed the exercise at fixed intensities.

The T2 group performed the exercise in a fixed final energy demand. To impose such a similar final demand within the T2 group, the total number of heartbeats for a running session was fixed. The selected number of beats to be attained was 6,300 (this number of beats resulted in sessions that lasted longer than the 30-min ones completed by the NR and T1 groups, but not so long as to compromise the similarity in the time of day for hormonal collection). From the preliminary data collection, the individual running time at R_65_ and R_80_ were computed for each volunteer as the session time = 6,300 / R_p_. As such, the mean running time at R_65_ was 46 minutes (± 4.1) min and 42 minutes (± 3.5) at R_80_.

### Running sessions

The experiments were conducted at a controlled room temperature of 21 (± 0.5) degrees Celsius. The average relative air humidity was 66% (± 10%). Estimated altitude of the laboratory: 785 meters above sea level.

All running sessions were performed in the morning, beginning between 08:00 and 09:00. There were two running sessions, one day at 65% of the heart rate reserve (R_65_) and the other at 80% of the heart rate reserve (R_80_). The order of the sessions was randomly assigned to each volunteer.

During the running session, the volunteer's heart rate was continuously monitored by surface ECG. The electrodes were attached to the thorax and abdomen (CM5 – a modified Lead I configuration). The ECG was acquired using a sampling rate of 1000 Hz and standard filters with a MP30 interface and the Biopac Student Lab Pro software (Biopac Systems Inc., Goleta, CA, USA).

### Hormone collection and analysis

The saliva samples for the hormonal analysis were obtained by direct salivation into plastic tubes, stored in a thermal bag at 10° Celsius and then in a freezer (at – 20° Celsius).

Samples were taken at three different occasions, at approximately the same time of day, in order to avoid circadian variations.

Basal hormones. Basal samples were collected at 08:00 and 11:00 on days when the volunteer did not perform any physical exercise. The volunteers were required to eat a small meal with around 40 grams of carbohydrate 1 hour before the first saliva sample (07:00).

Experimental hormonal data. On the experimental days, the saliva was collected prior to physical activity (around 08:00 – PRE; the volunteers were instructed to maintain a similar routine pattern as on the day of basal collection), immediately after the bout of exercise (around 08:45 – POST), and later on after the activity (around 11:00 – LATE). The volunteers conducted their normal daily activities between the POST and the LATE samples, but were oriented not to ingest large amounts of food.

Salivary cortisol and testosterone were measured using a specific enzyme-linked immunosorbent assay (ELISA) for each hormone (DiaMetra – Salivary Steroid Hormones, Italy; intra-assay variation ≤ 10% and inter-assay variation ≤ 8.3% for the cortisol kit, and intra-assay variation ≤ 8.0% and inter-assay variation ≤ 13.2% for the testosterone kit). Testosterone was measured in picograms per milliliter, and cortisol in nanograms per milliliter.

### Statistical approaches

First stage: In order to characterize the primary differences between the groups, a one-way ANOVA or two-way ANOVA with repeated measures was employed, as indicated in the results section. Statistical significance was set at p £ 0.05 in this stage.

Second stage: Heart rate. The question of whether the NR group has similar heart rates as the T1 and T2 groups as a whole was addressed by a t-test using the Holm-Bonferroni method for post-hoc significance. A priori, there were four potential comparisons: NR vs. T1; NR vs. T2, NR vs. (T1 and T2), T1 vs. T2. Therefore, the adjusted significance levels (critical p-values) were 0.013, 0.017, 0.025, 0.050 for the ordered p-values obtained in the comparisons.

Time-profile of the T:C ratio. In this case, for each group, there were three potential comparisons: PRE vs. POST; PRE vs. LATE; POST vs. LATE. In a similar manner to the heart rate comparisons, the differences were addressed by a t-test using the Holm-Bonferroni method for post-hoc significance. The adjusted significance levels were 0.017, 0.025, 0.050 for the ordered p-values obtained in the comparisons.

## RESULTS

The first step in the analysis was the characterization of the groups based on their heart rates in each running section (Table 2). A one-way ANOVA comparison of the heart rates showed an F-critical (2.15) of 3.68 and the F values obtained for the R_65_ and R_80_ were 5.53 and 3.72, respectively (p-values of 0.015 and 0.048), leading to the conclusion that the groups are dissimilar in their heart rates.

**Table 2:** Heart rates

Group	Subject	R_65_			R_80_		
		bpm	min	thb	bpm	min	thb
NR	1	150	30	4500	164	30	4920
	2	159	30	4770	169	30	5070
	3	145	30	4350	161	30	4830
	4	154	30	4620	163	30	4890
	5	140	30	4200	153	30	4590
	6	155	30	4650	160	30	4800
T1	1	146	30	4380	157	30	4710
	2	139	30	4170	156	30	4680
	3	133	30	3990	151	30	4530
	4	128	30	3840	141	30	4230
	5	123	30	3690	141	30	4230
	6	135	30	4050	156	30	4680
T2	1	140	45	6300	161	39	6300
	2	128	49	6300	140	45	6300
	3	146	43	6300	157	40	6300
	4	119	53	6300	131	48	6300
	5	140	45	6300	150	42	6300
	6	150	42	6300	158	40	6300

Heart rates (bpm), running time (min) and total heartbeats (thb) for each individual in each protocol (65% and 80%).

Comparisons between groups (see statistical approaches section) revealed that NR is different from the T group as a whole (p = 0.004 for R_65_, and p = 0.013 for R_80_), while T1 and T2 are not different (p = 0.596 for R_65_, and p = 0.887 for R_80_).

Therefore, despite the fact that the individuals in the NR group are not sedentary, their heart rates are higher than the those of individuals in the T1 and T2 groups, who had trained for running.

Table 3 contains the raw T:C ratio data for all the volunteers in the present study. A two-Way ANOVA with repeated measures detected that the 80% intensity zone generates a difference among the groups (detected as the groups themselves, p = 0.00004, and in the sample time, p = 0.01), while at the 65% level these differences are not significant.

**Table 3:** T:C data

Group	Subject	R_65_			R_80_		
		PRE	POST	LATE	PRE	POST	LATE
NR	1	11.40	17.67	13.41	13.58	13.17	25.38
	2	15.12	14.75	13.59	9.68	13.85	14.76
	3	17.14	12.11	18.71	13.25	8.48	22.50
	4	11.72	15.60	11.88	18.94	23.63	15.16
	5	20.80	19.31	22.58	16.87	23.46	23.64
	6	9.19	12.26	15.85	21.00	21.34	14.26
T1	1	10.47	15.68	9.14	12.92	14.69	10.00
	2	19.46	11.84	14.74	10.20	17.37	17.30
	3	14.89	18.06	13.00	13.13	16.45	13.51
	4	16.96	17.77	17.23	11.26	15.67	15.81
	5	15.92	20.05	22.39	13.35	15.27	18.13
	6	12.30	14.03	15.15	13.05	16.62	15.03
T2	1	9.09	13.32	12.36	21.40	23.23	15.84
	2	10.92	11.21	14.48	15.38	26.89	21.15
	3	9.31	17.20	16.60	13.37	23.43	24.07
	4	19.76	22.11	26.52	17.64	23.11	23.57
	5	9.67	13.37	18.43	18.47	22.15	23.32
	6	12.81	14.85	16.47	17.88	23.77	15.69

PRE: prior to running; POST: immediately after running; LATE: around 2.5 hours after the running section.

To complete the analysis and remain on track with the focus of the present study, i.e. whether there is a biphasic time-profile in the T:C ratio elicited by physical activity, the time-profile (i.e. PRE, POST and LATE) within each group was characterized as described in the statistical analysis section:

- Non-runner group: there were no differences over time both at the 65% and 80% intensity zones.
- T1 runner group: in the 80% intensity zone, POST > PRE (p = 0.0059).
- T2 runner group: in the 65% intensity zone, POST > PRE (p = 0.0231) and LATE > PRE (p = 0.0021), although POST and LATE were not different. In the 80% intensity zone, POST > PRE (p = 0.0083).

**Figure 2:**
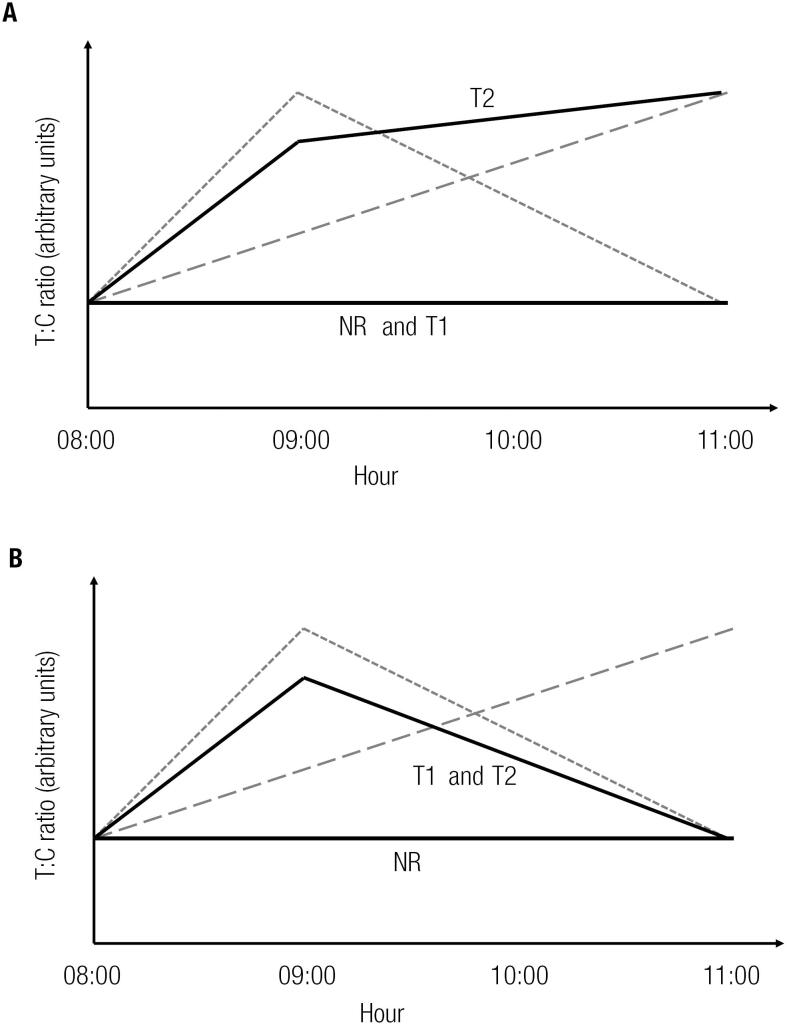
Graphical representation of the T:C results shown in Table 2. **(A)** Target heart rate of 65% (R_65_). **(B)** Target heart rate of 80% (R_80_). The curves shown in Figure 1 are in faded gray (circadian and biphasic).

Figures 2A and 2B are graphical representations of these results superimposed on the circadian and biphasic response hypothesis depicted in Figure 1.

## DISCUSSION

A biphasic time-profile in the testosterone:cortisol ratio can be seen in short-bout, high intensity exercise (treadmill running) during the morning in men trained for this specific physical activity. Irrespective of the level of training and intensity, it seems that a session of exercise in the morning causes a disruption in the circadian rhythm of the T:C ratio. How long such a disruption remains during the rest of the day deserves further investigations. Moreover, whether the modification in the T:C ratio promotes a catabolic milieu or not remains an open question, as well as the use of this ratio to evaluate the performance of athletes in acute physical exercise.

The results of the present study suggest that biphasic behavior in the T:C profile seems to depend on the energetic demand of the exercise, as well as the training status of the individuals. Therefore, in the 80% heart rate target, both groups of runners show the biphasic time-profile, while the non-runner group does not. However, at the 65% level, none of the groups present the hypothesized biphasic response.

This also answers our questions regarding whether there is an association between the biphasic time-profile and the intensity or duration of the acute physical exercise. The answer is yes, there is such an association between the biphasic time-profile and both intensity and duration, in the sense that biphasic behavior of the T:C ratio could only be detected in more elevated levels of demand, i.e. at the 80% heart rate target.

Our final query was in respect to the putative biphasic time-profile and the individual's training status. The results shown in Figure 2B indicate that training specificity plays a role in eliciting a biphasic response.

Here, in a biphasic mode or otherwise, and despite the specific training level, it seems that a bout of acute exercise is able to perturb the circadian rhythm of T:C, reducing the ratio found a few hours after physical activity. An exception for that was the T2 group in the 65% target, and we have no hypothesis or explanation for this result.

Tremblay and cols. (35) describe a significant increase in cortisol only after 120 minutes of running at 55% of ${\dot{\text{V}}}_{O2\max}$ in trained runners (contrasting with running sessions of 40 and 80 minutes). Jacks and cols. (36) evaluated the salivary cortisol responses in 60-minute exercise sessions on a cycle ergometer at three intensities (44.5, 62.3 and 76% of ${\dot{\text{V}}}_{O2\max}$ ) in 10 untrained men. The hormone fell in the low and moderate intensities and increased in the high intensity. Despite the intensity, cortisol sampled at 40 minutes during the bouts of exercise showed no significant difference (36). Hoffman (37) describes a dual response of testosterone depending on the duration of the physical activity, with an increase in short bouts (less than 2 ½ hours) and a reduction in exercises lasting longer than 3 hours. Therefore, the picture of a “rise and fall” in cortisol and testosterone level is not evident *prima facie*. Actually, many of these studies do not consider the biphasic time-profile and, as a consequence, additional data acquisition (e.g. at different post-exercise times) could lead to different results.

In a recent meta-analysis (33), the authors concluded that salivary cortisol, and particularly testosterone, are highly influenced by both the study design and sampling time. The present results seem to support this, as described above. In addition, the time (period of the day) of sampling the hormones should be taken into account due to the circadian variation of cortisol and testosterone, both in their circulating levels and in their response curves.
